# Porphyria cutanea tarda: a case report

**DOI:** 10.1186/s13256-018-1956-9

**Published:** 2019-01-21

**Authors:** Hanife Usta Atmaca, Feray Akbas

**Affiliations:** Istanbul Training and Research Hospital, Internal Medicine Department, University of Health Sciences, Istanbul, Turkey

**Keywords:** Porphyria cutanea tarda, Liver disease, Skin lesion

## Abstract

**Background:**

The porphyrias are a rare group of metabolic disorders that can either be inherited or acquired. Along the heme biosynthetic pathway, porphyrias can manifest with neurovisceral and/or cutaneous symptoms, depending on the defective enzyme. Porphyria cutanea tarda, the most common type of porphyria worldwide, is caused by a deficiency of uroporphyrinogen decarboxylase, a crucial enzyme in heme biosynthesis, which results in an accumulation of photosensitive byproducts, such as uroporphyrinogen, which leads to the fragility and blistering of sun-exposed skin.

Porphyria cutanea tarda is a condition that affects the liver and skin by reduction and inhibition of uroporphyrinogen decarboxylase enzyme in erythrocytes. Areas of skin that are exposed to the sun can generate blisters, hyperpigmentation, and, sometimes, lesions that heal leaving a scar or keratosis. Liver damage might present in a wide range of ways from liver function test abnormalities to hepatocellular carcinoma. The toxic effect of iron plays a role in liver damage pathogenesis.

**Case presentation:**

A 59-year-old Turkish man presented with hyperpigmented skin lesions, fatigue, and elevated ferritin level and liver function tests. He was diagnosed as having porphyria cutanea tarda after a clinical investigation and treated with phlebotomy.

**Conclusion:**

Porphyria cutanea tarda is a rare condition of the liver but it must be remembered in a differential diagnosis of liver disease with typical skin involvement to decrease morbidity and health costs with early treatment.

## Background

Porphyria cutanea tarda (PCT) is a condition that affects liver and skin by reduction and inhibition of hepatic uroporphyrinogen decarboxylase (UROD) enzyme activity. The disease has been classified into three subtypes. Type I PCT has decreased hepatic UROD activity and is found in a sporadic fashion without family history. Type II PCT is an autosomal dominant disorder with genetic mutations of the *UROD* gene causing decreased UROD activity in all tissues. Type III PCT is similar to type II with respect to familial occurrence, but erythrocyte UROD activity is normal [1]. Homozygous familial PCT is extremely rare and is known as hepatoerythropoietic porphyria (HEP). This type of PCT is much more severe and develops during childhood, while the familial and sporadic forms appear at mid to late adulthood [2].

The UROD enzyme is needed to metabolize certain body chemicals that are known as porphyrins. It is the fifth enzyme of heme biosynthesis and inverts to uroporphyrinogen protoporphyrin [3]. Low levels of functional UROD results in abnormal accumulation of specific porphyrins in the body, especially in blood vessels, liver, and skin. The prevalence of all PCT is 1:5000–1:70,000 [4–7]. The disorder usually starts after 30 years of age and childhood occurrence is rare. Although it is an acquired disease, it sometimes is genetic (autosomal dominant). Genetic enzyme deficiency is usually latent and does not present any symptoms. The etiology is different for each patient. Environmental factors might also play a role. The etiological factors cause a reduction or inhibition in UROD enzyme in the liver and lead to clinical signs. Those signs are seen when UROD levels decrease below 20% [8]. Leading environmental factors are alcohol usage and presence of hepatitis C or human immunodeficiency virus (HIV). Certain medications (cytochrome P-450 inhibitors) and estrogen are other etiological factors [9, 10]. Some studies show that tobacco smoking is also a risk factor for PCT. Some chemicals (for example, hexachlorobenzene) [11, 12], end-stage renal disease, and lupus are rarely found to be related to PCT. All these factors are thought to decrease hepcidin levels in the body and cause iron storage in the liver. However, in most cases, a relationship between symptoms and assumed etiological factors cannot be demonstrated. For example, alcohol intake clearly contributes to the development of the disease but the disease is not common in alcoholics. In most cases, three or more risk factors are present.

## Case presentation

A 59-year-old Turkish man presented with fatigue, loss of energy, and dark colored urine. When asked, he declared that hyperpigmentation occurred in his hands and face after exposure to sun since last year and sometimes those skin parts blistered and healed leaving a scar. He used to consume alcohol socially but since last year started to take alcohol on a daily basis. His medical history and family history were both unremarkable. He was a butcher and he consumed over 300 gr of meat on most days. He declared that his complaints exaggerated after consuming large amounts of meat.

**Fig. 1:**
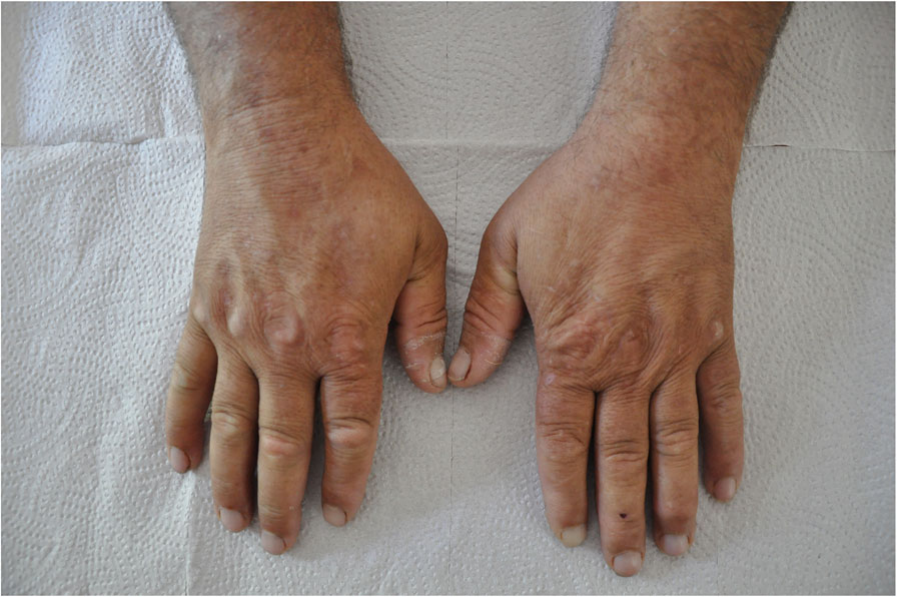
Skin involvement of porphyria cutanea tarda: Brown and white pigmented skin lesion on the dorsal surface of hands

A physical examination showed that both dorsal regions of his hands had brown pigmented skin lesions. A full skin examination revealed: erosions; scars; and 1-mm, firm, white papules consistent with milieu on the dorsal surface of his hands (Fig. 1). The skin color of his face was also dark and he declared that it happened 6 months ago. His body mass index (BMI) was 38 kg/m2. His pathological laboratory results were as follows: aspartate aminotransferase (AST) 125 U/L (normal 0–50), alanine aminotransferase (ALT) 89 U/L (normal 0–50), gamma-glutamyltransferase (GGT) 1190 U/L (normal 0–55), lactate dehydrogenase (LDH) 268 (normal 0–248), creatine kinase (CK) 174 U/L (normal 0–171), alkaline phosphatase (ALP) 123 U/L (normal 30–120), ferritin 503 ng/ml (normal 23–336), and vitamin B12 1275 pg/mL (normal 145–914). Hepatitis C, hepatitis B, and HIV tests were negative. Autoimmune screening was negative. Urine color was turbid.

His porphyrin (24-hour urine) was 832 μg/24 hours (normal ≤ 100); his porphobilinogen (24-hour urine) was 1.65 mg/24 hours (normal ≤ 1.65). We could not fractionate the urine porphyrins because we do not have the necessary equipment to measure carboxylate porphyrins (uroporphyrin and hepta-, hexa-, and pentacarboxyl porphyrin) in our hospital.

Our patient was diagnosed as having PCT according to his medical history, typical skin lesions, and supportive laboratory findings. Phlebotomy was started as treatment regimen (450 cc/every 2 weeks). After the sixth phlebotomy, his symptoms regressed and he declared that he felt better. His laboratory analysis also showed improvement (AST 30 U/L, ALT 38 U/L, and ferritin 43 ng/ml. A follow-up was started and an appointment to see him in 3 months was made.

## Discussion

In PCT, typical skin lesions, chronic liver disease, and symptoms resulting from iron overload are the most common signs of the disease. It usually presents with skin lesions. Skin parts that are exposed to the sun, such as the hands and face, are most commonly affected and patients have extreme photosensitivity. Easily bruised and blistered skin is common. Scars might develop or skin can get thicker and harder as seen in sclerosis, which in this case is called pseudosclerosis [13]. As commonly seen in patients with PCT, our patient also had atrophic and hyperpigmented skin lesions. There were no typical clinical signs or autoimmune laboratory findings for scleroderma. Thus we eliminated scleroderma in differential diagnosis. A lack of abdominal pain and neuropsychiatric findings of acute intermittent porphyria, discarded variegate porphyria and hereditary coproporphyria.

In patients with PCT, hepatic UROD activity is significantly reduced (mean 0.43 U/mg protein; range 0.25–0.99) as compared to normal individuals (mean 1.61 U/mg protein; range 1.27–2.42). Also, erythrocyte UROD activity is decreased in these patients. Iron overload in the liver causes liver damage. An oxidized form of uroporphyrinogen which is called uroporphomethene is an inhibitor decreasing UROD enzyme activity [14]. Uroporphomethene oxidation of uroporphyrinogen is iron dependent and it has an important role in PCT pathogenesis.

The UROD enzyme is vital to metabolize some chemicals in the body that are called porphyrins. Low functional UROD levels cause abnormal accumulation of porphyrins in blood, liver, and skin. PCT symptoms occur after abnormal accumulation of porphyrin and related chemicals. When they accumulate in the liver, they might cause toxic damage.

Liver involvement can present with iron overload in the liver (hepatic siderosis), fat overload in the liver (steatosis), and liver inflammation (portal triaditis and periportal fibrosis). Liver damage might present in a wide spectrum ranging from liver enzyme elevation to hepatic carcinoma. Our patient had a history of alcohol intake and his symptoms worsened after alcohol consumption. Alcohol increases iron absorption resulting in iron accumulation in the liver, it stimulates hepatic δ-aminolevulinate synthase (ALA) and free radical production and is independently hepatotoxic [15].

Clinical symptoms are usually related to abnormally elevated iron levels in the liver, yet the relationship between iron overload and PCT is not completely clear because there is not a certain hepatic iron level found to be related to PCT. Some patients with symptomatic PCT have normal iron levels. *HFE* gene mutation prevalence is increased in PCT [16, 17]. *HFE* gene mutation might cause hemochromatosis which is a disease characterized by iron accumulation in the body, especially in the liver. Hemochromatosis is related to low hepcidin levels which is the major factor in iron absorption and regulation of iron in the gastrointestinal system (GIS) and liver.

The diagnosis of PCT depends on the definition of characteristic symptoms, a detailed history, clinical evaluation, and some special tests (blood test, urine test, stool test and skin biopsy). An increase in uroporphyrin and heptacarboxyl porphyrin levels in urine is important for diagnosis. In our case, elevation in 24-hour porphyrin and porphobilinogen supported our diagnosis although they are not specific for the disease. Increased carboxylic porphyrins in urine is more specific (more increase in 8-carboxylic, with lesser increases in 7, 6, 5, and 4- carboxylic porphyrins) for diagnosis. In our case, their levels could not be measured due to lack of equipment in our hospital.

Familial PCT can be diagnosed by the presence of a low amount of UROD enzyme in erythrocytes. A diagnosis is made by testing the urine of the patient or a family member for porphyrins and/or analyzing UROD enzyme activity.

PCT treatment targets specific symptoms of patients. Treatment needs to involve a team of experts including general internists, hematologists, dermatologists, and hepatologists. PCT is the most benign type of porphyria and the treatment is effective for both familial and sporadic forms. The standard treatment of PCT is regular phlebotomy to decrease iron and porphyrin levels in the liver. The suggested approach is to make a phlebotomy of one whole blood unit (450 cc) every 2 weeks until the ferritin level reaches approximately 20 ng/mL [18, 19]. Independent from verified iron overload, this is the routine treatment in most porphyria centers. Phlebotomy is a safe and easy method of extraction of blood via blood vessels. As most of the iron in the body is in blood cells, regular phlebotomies decrease extreme iron levels. Regularly performed phlebotomies cause full remission in most cases. Usually 5–8 phlebotomies are needed to achieve full remission.

In some cases, patients can be treated with low-dose antimalarial drugs such as hydroxychloroquine or chloroquine, drugs that act as mobilizers of porphyrins from the liver, by transforming hepatocyte porphyrins into water-soluble complexes which are excreted in urine. This method is usually applied to patients who have anemia or blood vessel problems or do not want to have multiple phlebotomies. The dosage of the medication here is important. Actual doses might cause worsening of the photosensitivity and elevation of porphyrin levels. The suggested dose is 100 mg hydroxychloroquine twice a week or 125 mg chloroquine twice a week [20–22]. This regime is as effective as phlebotomy treatment, has lower cost, and is easier. There was no difference for treatment results between 2 × 100 mg chloroquine/week and 450 ml phlebotomy/2 weeks other than patients receiving chloroquine showed better compliance [23].

Iron chelators are iron binding drugs that cause iron to dissolve in water and get renally extracted. Iron chelators are less effective than phlebotomy or antimalarial treatments. However, they can be used in selected patients such as patients with end-stage renal disease on dialysis.

Patients with PCT are suggested to quit smoking tobacco and alcohol intake as these might trigger their disease. They should avoid direct exposure to the sun using a double layer of clothing, wide hats, gloves, and sun glasses. Orally administered analgesics might be used for painful skin lesions. It is important to prevent infection of skin lesions and, when it occurs, antibiotics need to be used. Full remission is possible with treatment of PCT but relapse is possible. Relapse treatment is the same as primary treatment.

## Conclusion

In our opinion, this case is rare because it was not diagnosed for a long time because the skin signs were indistinct at the beginning and elevated liver enzymes were considered a consequence of regular alcohol intake. PCT is a rare condition of the liver that is usually overlooked, but it must be remembered in a differential diagnosis of liver disease with typical skin involvement to decrease morbidity and health costs with possible early treatment. Correct definition of skin lesions in the presence of liver damage facilitates the diagnosis.

## References

[1] Sassa S. Modern diagnosis and management of the porphyrias. Br J Haematol. 2006;135(3):281–92. 10.1111/j.1365-2141.2006.06289.x 16956347

[2] Farrag MS, Mikula I, Richard E, Saudek V, De Verneuil H, Martásek P. Hepatoerythropoietic Porphyria Caused by a Novel Homoallelic Mutation in Uroporphyrinogen Decarboxylase Gene in Egyptian Patients. Folia Biol (Praha). 2015;61(6):219–26. 10.14712/fb2015061060219 26789143

[3] Anderson KE, Sassa S, Bishop DF, Desnick RJ. Disorders of Heme Biosynthesis: X-Linked Sideroblastic Anemia and the Porphyrias. In: Scriver CR, Beaudet AL, Sly WS, Valle D, editors. The Metabolic and Molecular Bases of Inherited Disease, vol. 2. New York: McGraw–Hill; 2001. p. 2991–3062.

[4] Chan OT, Tsai N, Wong RL, Izumi AK. The additive effects of hepatitis C infection and end-stage renal disease in porphyria cutanea tarda. Cutis. 2006;78(6):397–400. 17243426

[5] Frank J, Poblete-Gutierrez P. Porphyria cutanea tarda--when skin meets liver. Best Pract Res Clin Gastroenterol. 2010;24(5):735–45. 10.1016/j.bpg.2010.07.002 20955974

[6] Lim HW. Role of viral infection in porphyria cutanea tarda. Photodermatol Photoimmunol Photomed. 1997;13(3):75–7. 10.1111/j.1600-0781.1997.tb00116.x 9372518

[7] Sams H, Kiripolsky MG, Bhat L, Stricklin GP. Porphyria cutanea tarda, hepatitis C, alcoholism, and hemochromatosis: a case report and review of the literature. Cutis. 2004;73(3):188–90. 15074347

[8] Elder GH. Porphyria cutanea tarda and related disorders (Chapter 88). In: Kadish KM, Smith K, Guilard R, editors. Porphyrin Handbook, Part II, vol. 14. San Diego: Academic Press; 2003. p. 67–92.

[9] Wickliffe JK, Abdel-Rahman SZ, Lee C, Kormos-Hallberg C, Sood G, Rondelli CM, Grady JJ, Desnick RJ, Anderson KE. *CYP1A2*1F* and *GSTM1* alleles are associated with susceptibility to porphyria cutanea tarda. Mol Med. 2011;17(3–4):241–7. 10.2119/molmed.2010.00130 PMC3060985 20957336

[10] Wahlin S, Floderus Y, Stål P, Harper P. Erythropoietic protoporphyria in Sweden: demographic, clinical, biochemical and genetic characteristics. J Intern Med. 2011;269(3):278–88. 10.1111/j.1365-2796.2010.02236.x 20412370

[11] Schmid R. Cutaneous porphyria in Turkey. N Engl J Med. 1960;263:397–8. 10.1056/NEJM196008252630807 14442995

[12] Can C, Nigogosyan G. Acquired toxic porphyria cutanea tarda due to hexachlorobenzene. Report of 348 cases caused by this fungicide. JAMA. 1963;183:88–91. 14018209

[13] Mahmoud BH, Hexsel CL, Hamzavi IH, Lim HW. Effects of visible light on the skin. Photochem Photobiol. 2008;84(2):450–62. 10.1111/j.1751-1097.2007.00286.x 18248499

[14] Phillips JD, Bergonia HA, Reilly CA, Franklin MR, Kushner JP. A porphomethene inhibitor of uroporphyrinogen decarboxylase causes porphyria cutanea tarda. Proc Nat I Acad Sci USA. 2007;104:5079–84. 10.1073/pnas.0700547104 PMC1820519 17360334

[15] Bleasel NR, Varigos GA. Porphyria cutanea tarda. Australas J Dermatol. 2000;41(4):197–206. 10.1046/j.1440-0960.2000.00437.x 11105361

[16] Young LC. Porphyria cutanea tarda associated with Cys282Tyr mutation in *HFE* gene in hereditary hemochromatosis: a case report and review of the literature. Cutis. 2007;80:415–8. 18189029

[17] Egger NG, Goeger DE, Payne DA, *et al.* Porphyria cutanea tarda: multiplicity of risk factors including HFE mutations, hepatitis C, and inherited uroporphyrinogen decarboxylase deficiency. Dig Dis Sci. 2002;47:419–26. 10.1023/a:1013746828074 11855561

[18] Ratnaike S, Blake D, Campbell D, Cowen P, Varigos G. Plasma ferritin levels as a guide to the treatment of porphyria cutanea tarda by vene section. Australas J Dermatol. 1988;29(1):3–8. 10.1111/j.1440-0960.1988.tb01216.x 3250437

[19] Rocchi E, Gibertini P, Cassanelli M, Pietrangelo A, Borghi A, Ventura E. Serum ferritin in the assessment of liver iron overload and iron removal therapy in porphyria cutanea tarda. J Lab Clin Med. 1986;107(1):36–42. 3941293

[20] Malina L, Chlumský J. A comparative study of the results of phlebotomy therapy and low-dose chloroquine treatment in porphyria cutanea tarda. Acta Derm Venereol. 1981;61(4):346–50. 6173993

[21] Kordac V, Semrádová M. Treatment of porphyria cutanea tarda with chloroquine. Br J Dermatol. 1974;90(1):95–100. 10.1111/j.1365-2133.1974.tb06367.x 4811840

[22] Wolff C, Armas R, Krause P, Parraguez A, RamónSoto J. Treatment of porphyria cutanea tarda with chloroquine and its effect on associated liver disease: retrospective analysis. Rev Med Chil. 1996;124(4):456–60. 9110486

[23] Singal AK, Kormos-Hallberg C, Lee C, *et al.* Low-dose hydroxychloroquine is as effective as phlebotomy in treatment of patients with porphyria cutanea tarda. Clin Gastroenterol Hepatol. 2012;10:1402–9. 10.1016/j.cgh.2012.08.038 PMC3501544 22985607

